# How Much Gravity Is Needed to Establish the Perceptual Upright?

**DOI:** 10.1371/journal.pone.0106207

**Published:** 2014-09-03

**Authors:** Laurence R. Harris, Rainer Herpers, Thomas Hofhammer, Michael Jenkin

**Affiliations:** 1 Department of Psychology, York University, Toronto, ON, Canada; 2 Department of Computer Science, Hochschule Bonn-Rhein-Sieg, Sankt Augustin, Germany; 3 Department of Electrical Engineering and Computer Science, York University, Toronto, ON, Canada; Birkbeck, University of London, United Kingdom

## Abstract

Might the gravity levels found on other planets and on the moon be sufficient to provide an adequate perception of upright for astronauts? Can the amount of gravity required be predicted from the physiological threshold for linear acceleration? The perception of upright is determined not only by gravity but also visual information when available and assumptions about the orientation of the body. Here, we used a human centrifuge to simulate gravity levels from zero to earth gravity along the long-axis of the body and measured observers' perception of upright using the Oriented Character Recognition Test (OCHART) with and without visual cues arranged to indicate a direction of gravity that differed from the body's long axis. This procedure allowed us to assess the relative contribution of the added gravity in determining the perceptual upright. Control experiments off the centrifuge allowed us to measure the relative contributions of normal gravity, vision, and body orientation for each participant. We found that the influence of 1 g in determining the perceptual upright did not depend on whether the acceleration was created by lying on the centrifuge or by normal gravity. The 50% threshold for centrifuge-simulated gravity's ability to influence the perceptual upright was at around 0.15 g, close to the level of moon gravity but much higher than the threshold for detecting linear acceleration along the long axis of the body. This observation may partially explain the instability of moonwalkers but is good news for future missions to Mars.

## Introduction

Maintaining an upright posture in a low-gravity environment is not easy. NASA documents abound with examples of astronauts falling on the lunar surface [1], [2]. Even on the most recent moon visit (Apollo 17, 1972), Astronaut Harrison Schmidt fell over as he worked on the lunar surface [3]. The perception of the relative orientation of oneself and the world is fundamental not only to balance [4]–[9] but also for many other aspects of perception including recognizing faces and objects [10], [11], and predicting how objects are going to behave when dropped or thrown [12]. Indeed, recent emerging studies suggest that a functioning vestibular system may be required for depth perception [13], [14] and even for higher aspects of cognition such as the identity of self [15]. Misinterpreting the upright direction can lead to perceptual errors, for example misinterpreting the orientation of a vehicle, and can threaten balance if a person uses an incorrect reference orientation to stabilize themselves. It is therefore crucial to understand how the direction of up is established and to establish the relative contribution of gravity to this direction before journeying to environments with gravity levels different to that of Earth.

Establishing an “up” direction is a multisensory process that integrates information about orientation obtained from visual cues, gravity and the internal representation of the body [16]. Gravity typically contributes about 20% to the perceptual upright (PU: the direction in which polarized objects, including such things as writing, trees and people, are judged as being the correct way up) with the remainder coming from visual cues and the orientation of the body [17]. Many studies have estimated the threshold for detecting linear acceleration [18]. Estimates of this threshold vary considerably depending on the methods employed [19] but there is a general agreement that accelerations along the long axis of the body above about 0.15 m.s^−2^ (0.02 g) are reliably detectable. Recent studies using a limited set of g values in parabolic flight have suggested that much higher levels of g are needed to provide useable orientation cues [20], [21]. However, no systematic studies have investigated the threshold for the effect of maintained linear acceleration on a behavioural task. It is entirely unknown how much gravity is needed to establish a perceptual upright.

**Figure 1:**
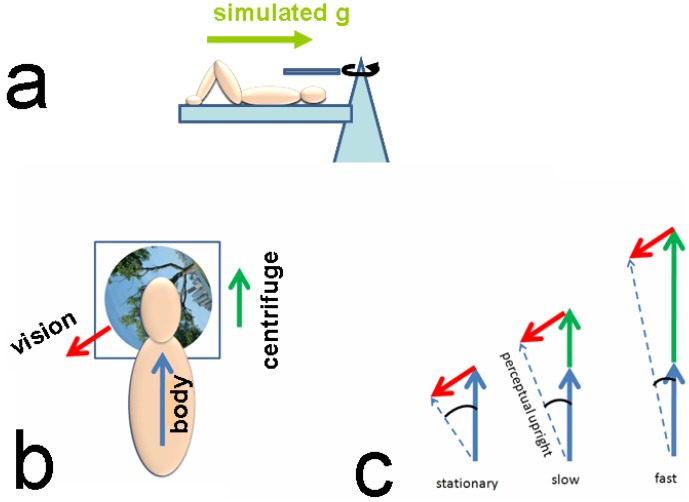
The experimental setup. (A) Participants lay on a human centrifuge with their feet out so that centripetal force from the centrifuge produced a centripetal force simulating gravity along the long axis of the body. (B) They viewed a screen mounted above their heads which presented a scene tilted at 112° relative to their bodies. The direction signaled by each cue to upright is indicated by arrow: red, vision; green, simulated gravity and blue, the body. (C) Thus, the three vectors involved in determining the perceptual upright (body, gravity and vision) could be dissociated.

To assess how much gravity is needed to establish an up direction, we had participants view a highly polarized visual scene while lying supine on a human centrifuge (Fig. 1a). We rotated the centrifuge at various speeds to create controlled, maintained linear accelerations along the long axis of the body (Fig. 1b). The visual scene they were viewing could be rotated about the naso-occipital axis, which had the effect of pulling the perceptual upright away from the body's axis towards the direction indicated by the visual background. As artificial gravity is added along the body's axis, there is a corresponding reduction in the relative influence of vision (Fig. 1c). This can be geometrically modeled and the effect of the added force can be plotted as a function of the amount of gravity added. Control experiments were done with no gravity in the coronal plane (by lying supine), lying on one side, and standing upright so that the relative contribution of body, gravity and vision could be assessed for each participant.

## Materials and Methods

### Participants

Ten participants (5 male, 5 female, average age 29.9±7.2 yrs) took part in the experiment. All participants were volunteers from the German Bundeswehr and had normal or corrected-to-normal vision and reported no history of vestibular dysfunction. Each participant was screened for diabetes mellitus, rheumatism, muscle or joint diseases, laser eye surgery, herniated disc, chronic back pain, claustrophobia, heart disease and orthostatic intolerance using anamnesis, clinical chemistry, urine analysis, ECG and through self report of general health. All experiments were approved by the Ethics Board of York University and the Ethics Commission of Aerztekammer Nordrhein (Duesseldorf, Germany). Participants gave their written informed consent and all experiments were performed in accordance with the principles expressed in the Declaration of Helsinki. During the centrifugation the following physiological signs were continuously monitored by a medical team: ECG, heart rate, SpO_2_, sphygmomanometric and finger blood pressure, and thoracic impedance. During the experiments, participants were also under continuous visual observation via an infrared camera system monitored by qualified medical personnel and the centrifuge operator.

### Centrifuge and associated screen

Experiments were run using the Short Arm Centrifuge Facility (SAHC) provided by the European Space Agency (ESA) situated at the Deutsches Zentrum für Luft-un Raumfahrt Institute of Medicine, in Cologne, Germany. Participants lay on their backs in a nacelle with their head towards the axis of rotation and with their knees bent to reduce the distance between the head and feet and hence the gravitational gradient along the body. They viewed an earth-horizontal screen (12×12.8 cm, 1024×768 @ 60 Hz) positioned 20 cm above their faces. The screen was mounted inside a lightproof hood to obscure the participant's view of the external environment; the room lights in the centrifuge room needed to remain on while the centrifuge was spinning for safety reasons. The screen was viewed through a circular aperture (diameter 12.2 cm, 34°) to avoid orienting cues from the edges of the screen.

Gravitational levels were calculated at the head, which was between 73 and 76 cm from the centre of rotation depending on the participant. Given the gravitational gradient, the force at the stomach would be roughly twice that at the head. For each participant, ten personalized rotation speeds were used to produce centrifugal accelerations of 0 (centrifuge stationary), 0.02, 0.04, 0.06, 0.08, 0.1, 0.2, 0.6 and 1 g. Rotation speeds were calculated for each participant to take into account small variations in the distance of each person's head from the axis of rotation. The centrifuge always span clockwise. Participants lay in the centrifuge with their legs bent as if they were sitting lying down which reduced the distance of the body extremities from the centre of rotation and hence reduced the gravity gradient along the body. Participants held an emergency stop button in their left hand mounted on the end of a hand grip which could be operated by their left thumb. Responses were made with their right hand.

The stimuli consisted of the character “p” (2° high) that could be presented at any orientation under control of an adaptive algorithm (PEST, [22]). The algorithm searched for the orientation at which the participants chose the interpretations “p” and “d” equally. The characters were presented on highly-polarized visual backgrounds that were tilted 112° to the left or right of the body midline. These orientations have been found to shift the perceptual upright maximally [17]. A third visual background of a neutral grey was also used to estimate the perceptual upright when visual orientation cues were not present.

### Off-centrifuge control apparatus

In order to compare the results using centrifuge-simulated gravity from real gravity we ran a set of off-centrifuge experiments using the same participants before and after their centrifugation. Three postures were used, upright (sitting on a chair), lying on the left side and lying on the right side in order to separate the orientation of the body and gravity to assess their relative effects (see [17]). The visual display was mounted on a flexible frame that could be positioned for comfortable viewing in all positions. The screen was always earth-vertical and the same background orientations were used as for the on-centrifuge experiments. The screen was viewed at 20 cm through a black circular shroud that masked the screen to a circle (34° diameter), obscured peripheral vision and acted as a semi-rigid, padded head restraint to control both the viewing distance and the orientation of the observer's head relative to the screen.

### Responding

For both centrifuge and ground conditions, participants responded by pressing one of two keys on a hand held Gamepad (Gravis Gamepad Pro) input device. Participants were instructed that two of the buttons corresponded to “p” and “d”. The centrifuge span so that the participant's right hand was facing into the wind and participants found this rather cold and so, for the centrifuge conditions, they wore a glove with the fingers cut out on their right, performing hand.

### Procedure

#### On-centrifuge

Participants lay on their back on the centrifuge bed with their knees bent and monitoring equipment for heart rate, breathing and blood pressure were connected. The hood containing the screen was positioned over their heads. A gravitational state was chosen pseudo-randomly from the list of states to be used (all participants ran the gravity conditions in the same sequence) and the machine was spun up to speed which took up to a minute depending the magnitude of the transition. Constant speed was maintained for 30 sec before the experiment began.

Once the steady state had been reached, participants viewed a background/character combination for 500 ms after which a grey screen of the same mean luminance appeared with a 0.45° diameter, central fixation spot. Following the disappearance of the stimulus, participants pressed a button to indicate which character they had seen: “p” or “d” (responses were not permitted while the stimulus was present). The next trial commenced after 150 ms. For a given background orientation, four PEST's were constructed initialized at 10° (restricted to the range 0° to 180°), 170° (restricted to the range 0° to 180°), 190° (restricted to the range 180° to 360°) and 350° (restricted to the range 180° to 360°) where 0° corresponds to the stem pointing towards the top of the screen (the “d” configuration). Participants were presented with these four PESTS x three visual stimuli in random order until all four PESTS terminated (after 13 reversals), which took between 10 and 17 minutes to complete for each body or centrifugal spin condition. Participants ran in four sessions, two separated by an interval of about an hour and then a complete repetition of the entire experiment a few days later. The two sessions contained the acceleration sequences [0 g, 0.02 g, 0.2 g, 0.08 g, 0.6 g] and [0.04 g, 0.1 g, 0.06 g, 1.0 g, 0 g]. Which sequence was run first was randomized. Off-centrifuge experiments were run before and after each complete centrifuge session.

#### Off-centrifuge controls

Participants were tested while sitting in an upright position and lying on their left and right side viewing a similar display to the one used on the centrifuge. The posture order was selected randomly for each participant. The same background picture was presented on the screen at the same three visual angles as on the centrifuge (relative to gravity).

### Data Analysis

For each combination of body tilt (upright, left side down, right side down) or centrifuge acceleration (from 0 to 1 g) and visual background (tilted left, tilted right or grey), the PSE indicated by each of the PESTs was obtained by averaging the value of the last three reversals. This gave two values for each “transition” between the “p” and “d” percept: one when the stem pointed into the left hemifield and one when it pointed into the left. The mean of the four PSE's was taken as the perceptual upright. These values were used to solve the vector geometry shown in figure 1b and described in the modeling section, below. Statistics were performed using t-tests. The database can be obtained from http://www.yorku.ca/harris/centrifuge.xls

## Results

### Off-centrifuge effects

**Figure 2:**
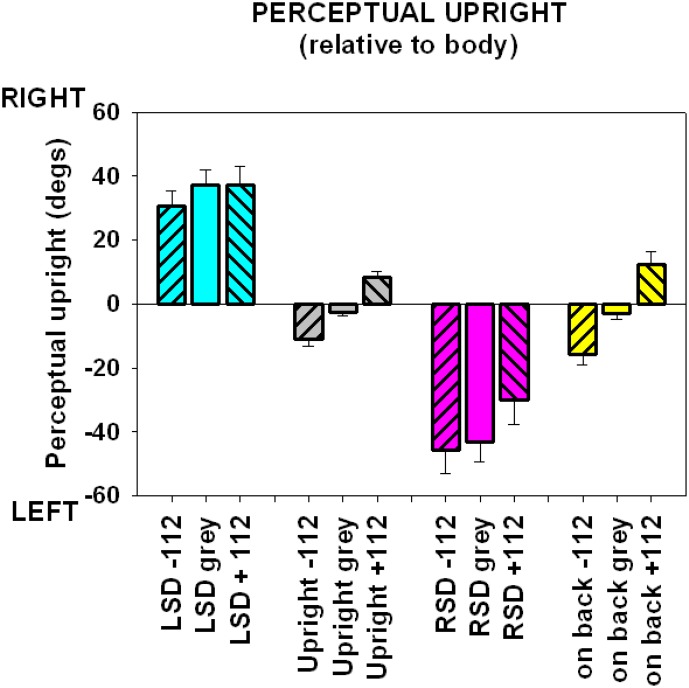
The variation of the perceptual upright in response to body posture (blue left side down, pink right side down, grey upright, yellow on back) and vision (left hash, 112° L; right hash 112° R; clear, grey background). Errors are standard errors.

In order to obtain the influence of vision and gravity on the perception of upright (PU) in our participants, we first obtained the direction of the PU with the directions signaled by vision, the body, and gravity separated by viewing a grey visual background or a background tilted 112° left or right relative to gravity with the person upright, on their side, and on their back (on the centrifuge before the centrifuge started to move). The average directions of the PU found for each of these variations are shown in Figure 2. The effect of the background was to tilt the perceptual upright in the direction of vision. When the body was tilted to the left, the PU shifted to the right (relative to the body, i.e., towards the direction of gravitational up) and visa versa. The purpose of this section was to ascertain baseline measures to compare with data collected when forces were applied along the long axis of the body by the centrifuge data (see modeling section, below).

### On-centrifuge effects

**Figure 3:**
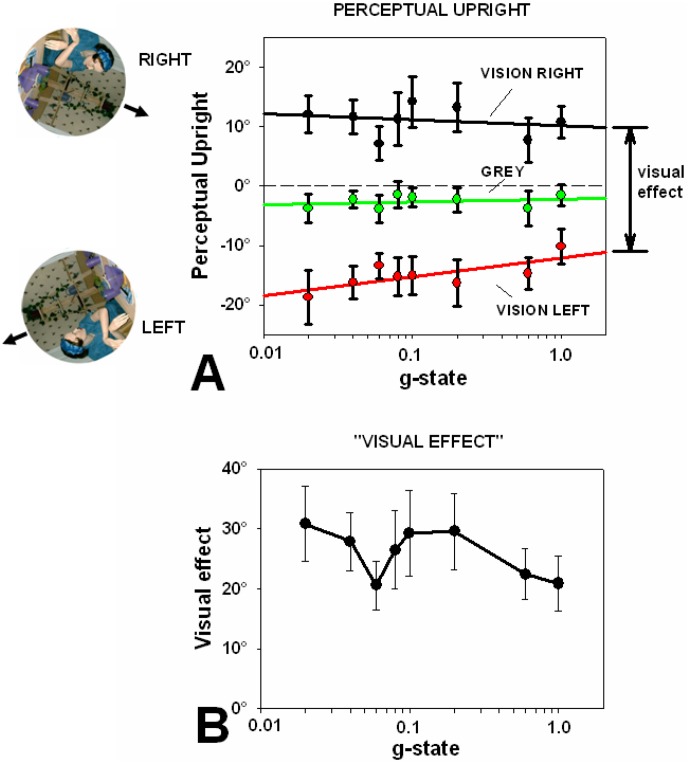
The effect of vision on the perceptual upright during accelerations from 0.02–1 g. Panel A shows that, on average, there was a decline in the effect of vision (less tilt away from the body midline, 0°) with increasing acceleration. In order to illustrate this better, the results with the visual background tilted left (red) were subtracted from those data collected when the visual background was tilted right (black) for each subject to obtain what we refer to as the “visual effect” (see Dyde et al., 2006). This is plotted in panel B.

Participants lay on a centrifuge (Fig. 1a) that was accelerated to various speeds evoking centripetal accelerations at the participant's head from 0 to 1 g. For each value, participants ran three conditions (vision tilted left, vision tilted right, and grey background). The perceptual upright under each of these conditions is shown in Fig. 3a averaged across participants.

The influence of the visual background was assessed by comparing the effect of tilting the background 112° right and left. The visual effect was defined as the difference between the perceptual upright measured with the background tilted by these amounts. A paired-samples t-test indicated that the visual effect supine (M = 28.01°, SD = 20.3°) was higher than the visual effect under 1 g centrifugation (M = 20.9°, SD = 14.5°), t(9) = 2.033, p = 0.036, d = 0.643. A one-tailed p-value is reported due to the strong prediction of a reduction in the VE. A paired-samples t-test indicated that the visual effect under 1 g centrifugation (M = 20.9°, SD-14.5°) was not significantly different than the visual effect when upright (M = 19.1°, SD = 11.2°), t(9) = 0.974 p = 0.355, n.s.

### Modeling

We have previously shown that the perceptual upright can be well predicted from a vector sum of vision, the body and gravity [17]. This model is illustrated in Fig. 1 to describe what was expected on the centrifuge. Since the participant's body was aligned with the centripetal force (as it is aligned with gravity when standing upright), we were unable to separate the effects of gravity and the body for each centrifugation condition. We made the assumption that the body and visual vectors remained constant throughout the experiment and only the gravity vector varied. We calculated the lengths of each of these vectors (relative to each other, as only relative measures can be obtained) from the off-centrifuge data using:
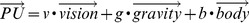
(1)


**Figure 4:**
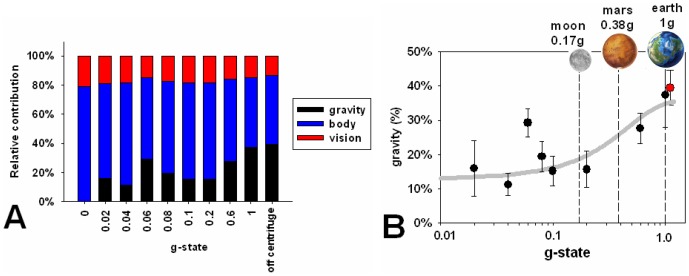
The effect of adding centrifugal force on the perceptual upright. (A) The relative contribution of each of the vectors corresponding to vision, body and gravity during centrifugation (see key). These are expressed as percentages, so the total always equals 100%. (B) The relative weighting of gravity (relative to vision + body) expressed as a percentage of the total (see Fig. 3b) is plotted as a function of the imposed g-state. Also shown, for reference, are the gravitational fields of the moon, mars and earth. Plotted through the data is a sigmoidal function, which suggests a threshold around 0.15 g. The red symbol is the value from the off-centrifuge experiments. The data at 0.06 g is inconsistent with the general trend found in participants' responses. Further work is required to determine if this is a statistical anomaly or represents a true but unusual response of our subject pool. Errors are SEM.

Where 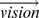, 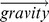 and 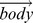 are the directions signaled by each cue, weighted by factors v, b and g respectively. A rotational bias term for the PU was also introduced. The ratio v∶b∶g assessed using normal gravity conditions was 14%∶47%∶39% which is similar to that reported in [17] (25%∶54%∶21%) although with more emphasis on gravity and less on vision in this population. People vary enormously in the relative weightings assigned to each vector but the individual weightings are constant over time for each person. Making the assumption that the relative weightings of vision and body remained constant (v/b = 0. 29 on average) for each person throughout their centrifuge experience, we then fitted equation 1 and obtained a relative weighting for gravity for each value of added centripetal acceleration. This is shown in Fig. 4.

## Discussion

We have shown that centripetal force applied along the long axis of the body is as effective at contributing to our sense of the perceptual upright as when standing with normal gravity (compare the red and black symbols at 1 g in Figure 4b). This is despite the fact that participants on a centrifuge actually experience a summation of the centripetal force and gravity (a combined acceleration of 13.9 m.s^−2^). The lack of effect of the component of gravity outside the plane of the screen is also supported by the larger visual effect found when participants were lying supine (without centrifuge movement) both here and in previous studies [17]. Our data suggest that a gravitational field of about 0.15 g is necessary to provide effective orientation information. This value is compatible with the results of studies that have varied g using parabolic flight [20], [21] and is close to the gravitational force on the Moon of 0.17 g.

The short-arm centrifuge generated a gravitational gradient along the long axis of the body. The force at the stomach was about twice what it was at the head in our set up. Some subjects may be more affected by the force at the level of the somatic graviceptors [23] than at the head [24]. If this were the case, then our estimates of thresholds could have been underestimated.

For whole-body linear acceleration, the vestibular threshold is around 0.1 m.s^−2^ (although studies have reported values ranging from 0.014 to 0.25 m.s^−2^ [18], [19], [25]) and so the lunar value of 1.6 m.s^−2^ should be well above threshold. These values are compatible with Homick and Miller's conclusion that lunar gravity is an adequate stimulus for the otolith organs to define a gravitational vertical and to guide posture control [26]. Their conclusion, however, was based on anecdotal reports from Apollo astronauts that they experienced no disorientation on the lunar surface [1]. Our quantitative assessment suggests otherwise. We find that, even when the simulated gravitational force was above the acceleration threshold, it was only effective at influencing the perceptual upright above about 0.15 g: indeed, the gravitational force on the moon would only barely be able to provide adequate gravitational cues necessary for orientation. This is in agreement with recent studies using microgravity [21] who found even higher thresholds averaging around 0.3 g. Interestingly, these authors suggested that there may be an age effect in which younger participants show lower thresholds. Our participants were around 30 yrs old which puts them in the younger age range of those tested by de Winkel et al. [21] where thresholds extremely comparable to the ones reported here were found. The discrepancy between physiological and functional thresholds is not surprising. The equivalent situation in the visual system would be trying to predict the amount of light needed to recognize, for example, a face (perception) from knowing the minimum amount of light that can be detected in a dark room (sensation). However, surprisingly, thresholds for the perceptual consequences of linear acceleration have never before been systematically investigated.

A lower contribution of gravity corresponds to a higher relative significance applied to vision. Such an increase has also been observed in medicated Parkinson's patients [27] and might partially account for the reported balance problems both for Parkinson's patients and that have been associated with arrival on the moon [28]. Extrapolating our results to the situation in space after adaptation to the microgravity environment (see [29] for review), must be done with caution. Benson [30] showed that 0.22 g was not adequate to provide a vertical reference during experiments in the International Microgravity Laboratory (IML-1) on board Spacelab and Clément et al. [31] showed that in microgravity, 0.5 g provided by a centrifuge was enough to produce a perceived tilt of 90°. These values suggest that indeed it is likely to be the case that even after adaptation to a microgravity environment, forces in excess of 0.15–0.3 g are required to provide a behaviourally useful gravitational reference. When the cues that define the perceived upright are misaligned, for example when the body or visual reference plane is tilted relative to gravity, an unusual pattern of sensory weightings, where gravity was weighted less than expected, could potentially pull the perceived direction of upright more in the direction of the relatively higher weighted cues and thus threaten the reliability of processes that rely on the perceptual upright.

## Conclusion

We conclude that human centrifugation is a valid tool for investigating the effect of gravity on perceptual processes: 1 g applied along the long axis of the body by centrifugation produces comparable effects in the plane in which it was applied as 1 g applied by standing. We find a large discrepancy between physiological and functional thresholds from which we recommend caution when preparing for exposure to low gravity fields.
